# An Aqueous-Ethanol Extract of *Liriope spicata* var. *prolifera* Ameliorates Diabetic Nephropathy through Suppression of Renal Inflammation

**DOI:** 10.1155/2013/201643

**Published:** 2013-08-20

**Authors:** Hung-Jen Lu, Thing-Fong Tzeng, Jui-Chin Hsu, Shih-Hao Kuo, Chia-Hsin Chang, Shin-Ya Huang, Fang-Yu Chang, Ming-Chang Wu, I-Min Liu

**Affiliations:** ^1^Department of Food Science, College of Agriculture, National Pingtung University of Science and Technology, Neipu Township, Pingtung County 91201, Taiwan; ^2^Department of Pharmacy & Graduate Institute of Pharmaceutical Technology, Tajen University, Yanpu Township, Pingtung County 90701, Taiwan

## Abstract

The tuberous root of *Liriope spicata* var. *prolifera* (TRLS; Liliaceae family) is valued for the ability to promote glucose homeostasis, and it may therefore be utilized as an adjuvant therapy in the control of diabetic complications. The aim of the present study was to examine the effects of an aqueous ethanol extract from TRLS (TRLS-ext) (100 or 200 mg kg^−1^ per day for eight weeks) on rats with streptozotocin-induced diabetic nephropathy (DN). Renal dysfunction in diabetic rats was ameliorated by TRLS-ext as evidenced by reduced creatinine clearance, as well as increased blood urea nitrogen and proteinuria. Treatment with TRLS-ext was found to markedly improve histological architecture in the diabetic kidney. Hyperglycemia induced degradation of inhibitory kappa B and reduced nuclear factor kappa B activation, leading to increased infiltration of macrophages and increased levels of proinflammatory cytokines, including interleukin-1 and tumor necrosis factor-**α**. All of the above abnormalities were reversed by TRLS-ext treatment, which also decreased the expression of intercellular adhesion molecule-1, monocyte chemoattractant protein-1, and fibronectin in the diabetic kidneys. These findings provide a perspective on the renoprotective effects of TRLS-ext in DN.

## 1. Introduction

Diabetic nephropathy (DN), a major long-term complication of diabetes mellitus, is the most common worldwide cause of end-stage renal disease requiring dialysis [1]. It presents a staggering challenge to public healthcare systems due to the prohibitive cost of renal replacement therapy, which may become unaffordable even in developed countries.

Although DN is traditionally considered a nonimmune disease, accumulating evidence now indicates that immunologic and inflammatory mechanisms play significant roles in its development and progression. Diverse cells and proteins are implicated in the development of DN: leukocytes, monocytes, and macrophages; chemokines (e.g., monocyte chemoattractant protein-1, MCP-1); adhesion molecules (e.g., intercellular adhesion molecule-1, ICAM-1); inflammatory cytokines, such as interleukin-1 (IL-1) and tumor necrosis factor-**α** (TNF-**α**); and nuclear factors (e.g., nuclear factor kappa B; NF-*κ*B) [2, 3]. Genetic and pharmacological approaches that reduce inflammation in DN have not only enhanced our understanding of the disease pathophysiology, but also shown promise as potential therapeutic strategies.

The tuberous root of *Liriope spicata *var. *prolifera* (TRLS; Liliaceae family), commonly known as “maidong” in traditional Chinese medicine, has been used to treat cough and heart diseases and to be a substitute for the official crude drug Ophiopogon japonicus (Thunb.) Ker-Gawl for centuries [4]. Because of its high availability and safety, it is regarded as both food and medicine by the Chinese Ministry of Public Health. Previous phytochemical investigations with TRLS have revealed a rich diversity of chemicals, including steroidal saponins, polysaccharides, and butyl fructopyranoside [5–7]. Water extracts and crude polysaccharides from TRLS have been reported to have hypoglycemic effects, markedly activating insulin signal transduction in type 2 diabetic mice [8, 9]. TRLS is valued for its ability to promote glucose homeostasis, and it may be used as an adjuvant therapy in the control of diabetic complications. However, the possibility that TRLS could prove beneficial in ameliorating diabetic renal damage has not been previously explored. 

Plants have played a major role in the introduction of new therapeutic agents [10]. In our opinion, randomly searching plants for new therapeutic agents is inefficient, and a selective search based on traditional knowledge would be more focused, more economic, and more productive. Analyses of renal biopsies from type 1 and type 2 diabetic patients who develop DN indicate that inflammatory infiltrates are similar in both groups [11], which is consistent with studies in diabetic animal models [12, 13]. In the present study, we used the streptozotocin-(STZ-) induced rat DN model to evaluate the potential of an aqueous ethanol extract of TRLS to inhibit the progression of DN and to investigate the possible underlying mechanism of action.

## 2. Materials

### 2.1. Plant Material and Extraction

TRLS was purchased from a local market in Pingtung County, Taiwan, on May 2012. The plants were identified by Professor Hong T. Y. (Department of Biotechnology, College of Pharmacy and Health Care at Tajen University). Random amplified polymorphic DNA analysis of TRLS was performed to identify DNA polymorphisms. The voucher specimen (lot no. LS20120523) has been deposited in our laboratory. TRLS (10 kg) was extracted twice in 100 L boiling distilled water. The extracts were combined and concentrated to dryness under vacuum. The residue was dissolved in water, diluted in ethanol to a final concentration of 75%, and left overnight. The supernatant was applied to a D101 resin chromatography column and eluted with 70% ethanol. The eluate was collected and concentrated under vacuum to give 45 g of extract (TRLS-ext). Doses refer to grams of this dry powder. TRLS-ext (20 g) was applied to a silica gel column and eluted with chloroform : methanol (5 : 1), which was evaporated to give a dry fraction. After repeated chromatography over silica gel and a Sephadex LH-20 column with the eluent chloroform : methanol : water (65 : 20 : 5), we obtained ruscogenin (about 98 mg) and ophiopogonin D (about 20 mg).

### 2.2. Animal Models

Male Wistar rats (8 to 10 weeks of age, 200–250 g) were obtained from the Animal Center of National Cheng Kung University Medical College. To induce diabetes, rats were given a single intravenous injection of 60 mg/kg streptozotocin (STZ; Sigma-Aldrich, Inc., St. Louis, MO, USA). Animals were considered to be diabetic if they had plasma glucose concentrations of 350 mg dL^−1^ or greater, in addition to polyuria and other diabetic features. All studies were carried out two weeks after the injection of STZ. All animal procedures were performed according to the Guidelines for the Care and Use of Laboratory Animals of the National Institutes of Health (United States), as well as the guidelines of the Animal Welfare Act. The study was conducted with the approval of the Institutional Animal Care and Use Committee (IACUC) at Tajen University (approval number: IACUC 99-24; approval date: December 23, 2011).

### 2.3. Treatment Protocols

STZ-diabetic rats in the treatment group were dosed with 100 or 200 mg kg^−1^ TRLS-ext in distilled water (1.5 mL kg^−1^) by oral gavage once daily for eight weeks. The dosage regime was selected based on a previous report demonstrating that an aqueous extract of TRLS at 100 and 200 mg kg^−1^ was potentially effective in improving hyperglycemia in diabetic mice [8]. Vehicle-treated groups of STZ-diabetic rats and normal rats were given 1.5 mL kg^−1^ distilled water by oral gavage over the same period. Animals had free access to standard rat diet (Harlan Teklad, Madison, WI, USA; Cat. no. 2018) and water throughout the entire treatment period. At the end of the eight-week treatment, the rats were weighed, and blood samples were collected from a tail vein. TRLS-ext treatment was continued even though the plasma glucose of STZ-diabetic rats was lower than 350 mg dL^−1^ during the eight-week treatment period. The evening prior to blood sample collection, animals were restricted to 3 g of chow (given at 18:00 h), which was consumed immediately, and thereafter had access to only water. The animals were transferred to metabolic cages (Shineteh Instruments Co., Ltd, Taipei, Taiwan), and urine was collected for 24 hours under a layer of toluene (to inhibit bacteria growth) and stored at 4°C for later analysis. Toluene had no detectable effect on the estimation of albumin and creatinine in the urine samples. Following urine collection, rats were sacrificed using an intraperitoneal injection of sodium pentobarbital (50 mg kg^−1^). 

The kidneys were dissected, rinsed with cold isotonic saline, and weighed. An index of renal hypertrophy was estimated by comparing the wet weight of the left kidney to total body weight. The cortical tissues from the right kidney were stored immediately at −80°C in liquid nitrogen for biochemical determinations and Western blot analyses. Other kidney tissues were fixed in 10% neutralized formalin for histology and immunohistochemistry.

### 2.4. Blood Sampling and Analysis

Blood samples were centrifuged at 2 000 ×g for 10 minutes at 4°C, and plasma was divided into aliquots for subsequent analyses. Plasma glucose concentration was determined using a diagnostic kit from BioSystem (Barcelona, Spain; Cat. no. COD12503). Serum creatinine (Scr) concentration was determined using a commercial assay kit purchased from Diagnostic Chemicals Limited (Connecticut, USA; Cat. no. 221-30). Blood urea nitrogen (BUN) was determined by kinetic reagent (Diagnostic Chemicals Limited, Cat. no. 283-30). Commercial enzyme-linked immunosorbent assay kits were used to quantify glycosylated hemoglobin (HbA_1c_) levels (Integrated Bio Ltd., Taipei, Taiwan; Cat. no. CSB-E08140r). All analyses were performed in accordance with the instructions provided by the manufacturers.

### 2.5. Analysis of Urine Parameters

The 24-hour urine samples collected from each diabetic rat and age-matched control were centrifuged at 2 000 ×g for 10 minutes. Urinary albumin concentrations were measured with the Nephrat II ELISA kit (Exocell, PA, USA; Cat. no. NR002). The concentration of creatinine in pooled urine samples was determined using a commercial assay kit (Diagnostic Chemicals Limited; Cat. no. 221-30). All analyses were performed in accordance with the manufacturer's instructions. Creatinine clearance (Ccr) was calculated in individual rats using the relationship Ccr = urine creatinine × (urine volume/plasma creatinine) × time [14]. 

### 2.6. Renal Histological Analysis

Renal tissues were fixed with 10% neutral formalin phosphate buffer, dehydrated through a graded alcohol series, embedded in paraffin, cut into 4 *μ*m sections, and stained with periodic acid-Schiff (PAS). The sections were examined with light microscopy by an experienced pathologist, and micrographs from six glomeruli were analyzed randomly at 400x magnification. Forty glomeruli in each kidney were graded with respect to the level of damage: 0, no lesion; 1, <25% sclerosis; 2, 26–50% sclerosis; 3, 51–75% sclerosis; and 4, >75% sclerosis. The glomerulosclerosis indexes were calculated using the following formula: glomerulosclerosis index = (1 × *n*_1_) + (2 × *n*_2_) + (3 × *n*_3_) + (4 × *n*_4_)/(*n*_0_ + *n*_1_ + *n*_2_ + *n*_3_ + *n*_4_), where *n*_*x*_ is number of glomeruli in each grade of glomerulosclerosis as reported previously [15]. Tubulointerstitial damage was defined as tubular necrosis, inflammatory cell infiltrate, tubular lumen dilation, or tubular atrophy. Damage was scored on a scale of 0 to 4: 0 = normal; 0.5 = small focal areas; 1 = involvement of less than 10% of the cortex; 2 = 10–25% involvement of the cortex; 3 = 25–75% involvement of the cortex; 4 = extensive damage involving more than 75% of the cortex [16].

### 2.7. Immunohistochemistry

Formalin-fixed, paraffin-embedded kidney tissue sections were used for immunohistochemical staining. After deparaffinization and hydration, the slides were washed in Tris-buffered saline (TBS; 10 mmol L^−1^ Tris HCl, 0.85% NaCl, pH 7.2). Endogenous peroxidase activity was quenched by incubating the slides in methanol and 0.3% H_2_O_2_ in methanol. After overnight incubation with mouse monoclonal anti-rat monocyte/macrophage antibody (anti-ED-1) (Santa Cruz Biotechnology Inc. CA, USA; Cat. no. sc-59103) at 4°C, the slides were washed in TBS. Horseradish peroxidase-conjugated goat anti-mouse secondary antibody was then added, and the slides were incubated at room temperature for an additional 1 hour. The slides were washed in TBS, incubated with diaminobenzidine tetrahydrochloride as the substrate, and counterstained with hematoxylin. A negative control without primary antibody was included in the experiment to verify antibody specificity. Intraglomerular ED-1-positive cells were counted in 200 glomeruli per group. For assessment of interstitial infiltration, positively stained cells located in the interstitial area were counted from more than 20 random cortical fields in each section, and the numbers were averaged for each section.

### 2.8. Cytosolic and Nuclear Extract Homogenization

The kidney was cut, frozen immediately in liquid nitrogen, and stored at −80°C until use. The frozen kidney was ground to a powder, mixed in ice-cold HEPES buffer (10 mmol L^−1^ HEPES, 0.2% Triton X-100, 50 mmol L^−1^ NaCl, 0.5 mmol L^−1^ sucrose, 0.1 mmol L^−1^ EDTA, and protease and phosphatase inhibitors), and homogenized with an ice-chilled Dounce homogenizer at 4°C. The mixture was centrifuged at 8 000 ×g for 10 min, and the supernatant was divided into aliquots and stored at −80°C as the cytosolic extract. The pellet was suspended in ice-cold buffer (10 mmol L^−1^ HEPES, 500 mmol L^−1^ NaCl, 10% glycerol, 0.1 mmol L^−1^ EDTA, 0.1 mmol L^−1^ EGTA, 0.1% IGEPAL, and protease and phosphatase inhibitors), vortexed for 15 minutes at 4°C, and centrifuged for 10 minutes at 10 000 ×g. The resulting supernatant was divided into aliquots and stored as the nuclear extract at −80°C. The purity of the extracts was confirmed by the absence of cross-reactivity with *β*-actin in Western blots. Small aliquots of cytosolic and nuclear extracts were stored at 4°C for protein estimation [17].

### 2.9. Western Blotting

Cytosolic (70 *μ*g total protein) and nuclear extracts (50 *μ*g total protein) were separated on a 7.5% to 15% polyacrylamide gel and transferred electrophoretically to nitrocellulose membranes. The membranes were blocked with 5% nonfat dry milk in Tris-buffered saline Tween 20 (TBST) (20 mmol L^−1^ Tris, pH 7.6, 137 mmol L^−1^ NaCl, and 0.1% Tween 20) for three hours at room temperature, followed by an overnight incubation at 4°C with polyclonal antibodies against rat ICAM-1 (Santa Cruz Biotechnology, Inc.; Cat. no. sc-1511), MCP-1 (Santa Cruz Biotechnology, Inc.; Cat. no. sc-1785), TNF-*α* (Santa Cruz Biotechnology, Inc.; Cat. no. sc-1350) and IL-1*β* (Santa Cruz Biotechnology, Inc.; Cat. no. sc-7884), inhibitory kappa B I*κ*B*α* (Santa Cruz Biotechnology, Inc.; Cat. no. sc-371), NF-*κ*B p65 (Santa Cruz Biotechnology, Inc.; Cat. no. sc-109), or *β*-actin (Santa Cruz Biotechnology, Inc.; Cat. no. sc-130656). All antibodies were used at a dilution of 1 : 1000. After washing three times with TBST, incubation with appropriate horseradish peroxidase-conjugated secondary antibodies was performed for 1 hour at room temperature. After three additional washes with TBST, the immunoreactive bands were visualized by enhanced chemiluminescence (Amersham Biosciences, Buckinghamshire, UK) according to the manufacturer's instructions. The levels of *β*-actin were estimated to check for equal loading of samples. Films were scanned and band densities were quantified by densitometric analysis using ATTO Densitograph Software (ATTO Corp., Tokyo, Japan). Renal cortical sections were sampled from four independent experiments.

### 2.10. Statistical Analysis

The results are presented as the mean ± standard deviation (SD) for each group of animals at the number (*n*) indicated. Statistical analysis was performed with one-way analysis of variance (ANOVA). The Dunnett range post hoc comparisons were used to determine the source of significant differences where appropriate. The renal morphohistology and the morphologic analysis for PAS staining were analyzed statistically using the Kruskal-Wallis Test and Dunn's Multiple Comparisons Test. Values of *P* < .05 were considered statistically significant.

## 3. Results

### 3.1. Effects of TRLS-ext on Plasma Glucose Level and Indices of Renal Function

After 7 days of administration, two doses of TRLS-ext (100 and 200 mg kg^−1^) reduced fasting blood glucose (FBG) levels of STZ-diabetic rats by 6.3 ± 2.8 and 10.5 ± 2.2%, respectively (data not shown). FBG levels were remarkably (*P* < .05) less than diabetic control group when STZ-diabetic rats were treated with TRLS-ext (100 and 200 mg kg^−1^) for 28 days (a fall of 14.5 ± 3.4 and 17.6 ± 2.9%, resp.) (data not shown). Treatment of STZ-diabetic rats for eight weeks with daily doses of TRLS-ext produced obvious blood glucose-lowering effects: 100 mg kg^−1^, 20.1 ± 3.2% reduction; 200 mg kg^−1^, 29.3 ± 3.7% reduction (Table 1). 

The mean HbA_1c_ level in STZ-diabetic rats was markedly higher (14.19 ± 2.81%) than that in normal rats (4.82 ± 1.16%, Table 1). Daily treatment with TRLS-ext at doses of 100 and 200 mg kg^−1^ for eight weeks decreased the levels of HbA_1c_ in STZ-diabetic rats by 17.9 ± 3.3% and 23.7 ± 2.8%, respectively, relative to the values in nontreated STZ-diabetic rats (Table 1).

STZ-diabetic rats showed an increase in 24-hour urine volume, accompanied by increase in urine protein excretion (Table 1). After eight weeks of TRLS-ext treatment, 24-hour urine volume and 24-hour urine protein excretion for STZ-diabetic rats were markedly less than those of their vehicle-treated counterparts (Table 1). In addition, Scr and BUN levels in STZ-diabetic rats were obviously higher than these in rats from the normal control group. These levels were effectively reduced in STZ-diabetic rats treated for eight weeks with TRLS-ext relative to levels in their vehicle-counterparts (Table 1). In particular, increased Ccr in STZ-diabetic rats was observed after eight weeks of TRLS-ext treatment (Table 1). 

### 3.2. Effects of TRLS-ext on Renal Morphology

At the end of the eight-week treatment, the mean weight of the left kidney and the ratio of kidney weight to body weight in STZ-diabetic rats were increased significantly compared with those in the normal group (*P* < .01). Treatment of STZ-diabetic rats with 100 mg kg^−1^ per day TRLS-ext slightly reduced the degree of renal hypertrophy (Table 1), while both kidney hypertrophy and the ratio of kidney weight to body weight were markedly (*P* < .01) reduced in STZ-diabetic rats treated for eight weeks with 200 mg kg^−1^ per day TRLS-ext (Table 1).

**Figure 1:**
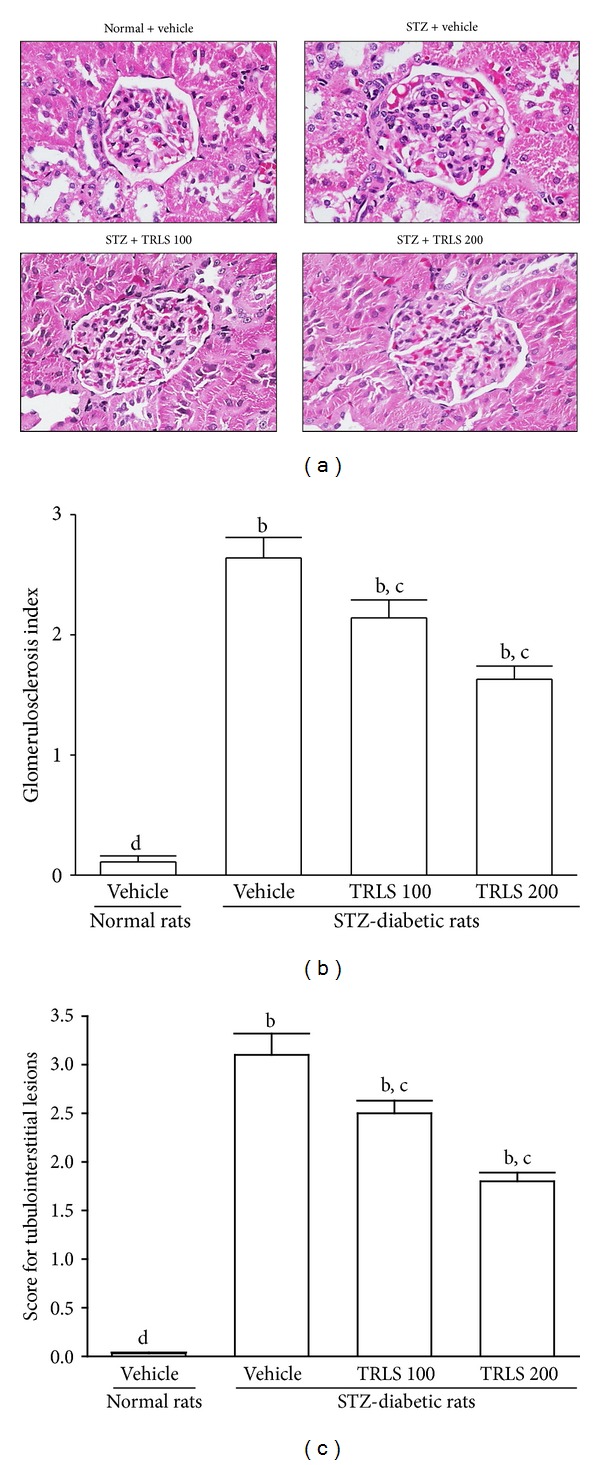
(a) Representative photomicrographs (original magnification, 400x) of PAS-stained kidney sections from STZ-diabetic rats treated for eight weeks with TRLS-ext. STZ-diabetic rats were dosed by oral gavage once daily for eight weeks with 100 mg kg^−1^ TRLS-ext (STZ + TRLS 100) or 200 mg kg^−1^ TRLS-ext (STZ + TRLS 200). Normal (normal + vehicle) or STZ-diabetic rats receiving vehicle treatment (STZ + vehicle) were administered the same volume of vehicle (distilled water) used to disperse TRLS-ext. (b) Results of quantification of the glomerulosclerosis index. (c) Score for tubulointerstitial lesions for each group. Values (mean ± SD) were obtained for each group of 4 animals. ^b^ *P* < .01 compared to vehicle-treated normal rats. ^c^ *P* < .05 and ^d^ *P* < .01 compared to vehicle-treated STZ-diabetic rats, respectively.

STZ-induced diabetes was accompanied by histopathological changes in renal tissues, including the expansion of mesangial matrix and, to a mild extent, thickening of glomerular basement membrane (Figure 1(a)). After eight weeks of TRLS-ext treatment, enlargement of the mesangia in glomeruli was mildly attenuated in the diabetes-affected renal tissues (Figure 1(a)). The glomerulosclerosis index for each group was shown in Figure 1(b). The score for tubulointerstitial lesions in each group was included in Figure 1(c).

### 3.3. Effects of TRLS-ext on Renal Macrophage Infiltration

**Figure 2:**
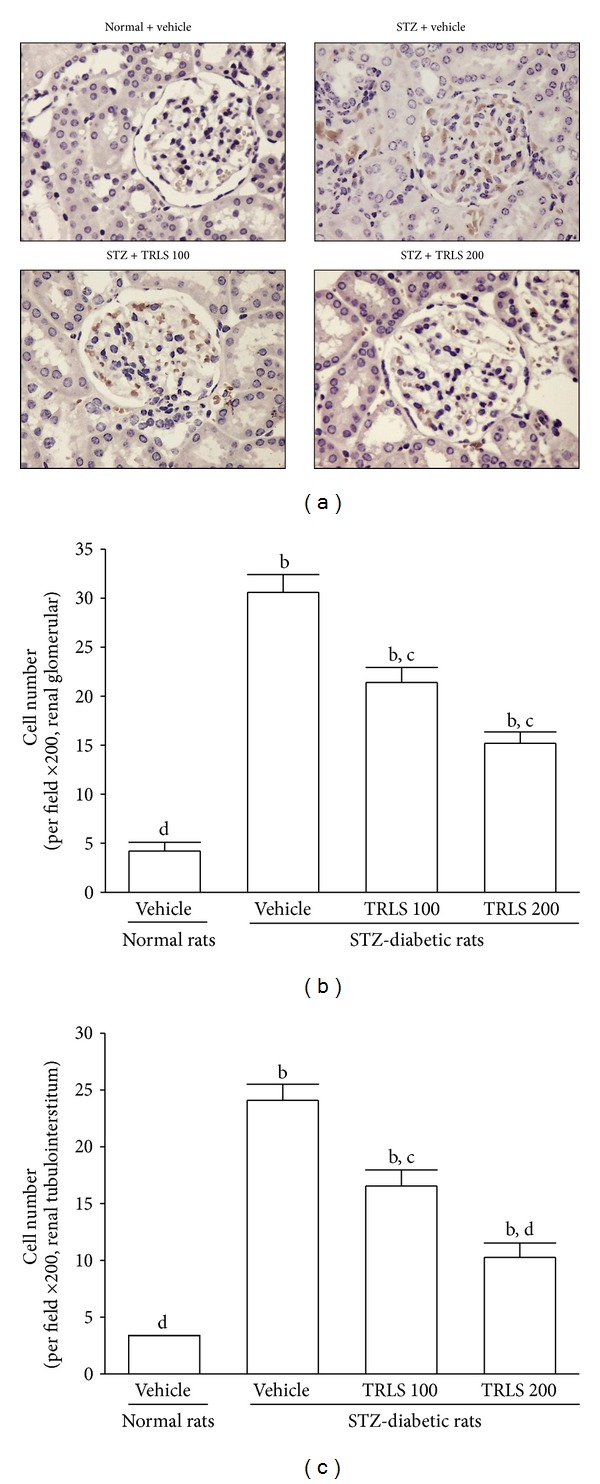
(a) Immunohistochemical staining (original magnification, 200x) for macrophage (ED-1-positive) cells in the renal tissues of STZ-diabetic rats treated for eight weeks with TRLS-ext. STZ-diabetic rats were dosed by oral gavage once per day for eight weeks with 100 mg kg^−1^ TRLS-ext (STZ + TRLS 100) or 200 mg kg^−1^ TRLS-ext (STZ + TRLS 200). Normal (normal + vehicle) or STZ-diabetic rats receiving vehicle treatment (STZ + vehicle) were given the same volume of vehicle (distilled water) used to disperse TRLS-ext. Quantified results are shown for number of macrophages in (b) renal glomerular compartments and (c) tubulointerstitium. Values (mean ± SD) were obtained for each group of 4 animals. ^b^ *P* < .01 compared to vehicle-treated normal rats. ^c^ *P* < .05 and ^d^ *P* < .01 compared to vehicle-treated STZ-diabetic rats, respectively.

Kidneys from control rats showed no significant macrophage infiltration (Figure 2). In contrast, prominent macrophage (ED-1-positive cells) infiltration was evident in the renal glomeruli and tubulointerstitium of STZ-diabetic rats (Figure 2). Treatment of STZ-diabetic rats with 200 mg kg^−1^ per day TRLS-ext for eight weeks caused a 50.2% and 58.3% reduction of macrophage influx in renal glomeruli and tubulointerstitium of STZ-diabetic rats relative to that in their vehicle-treated counterparts (Figure 2).

### 3.4. Effect of TRLS-ext on Renal ICAM-1, MCP-1, and Fibronectin Protein Expression

**Figure 3:**
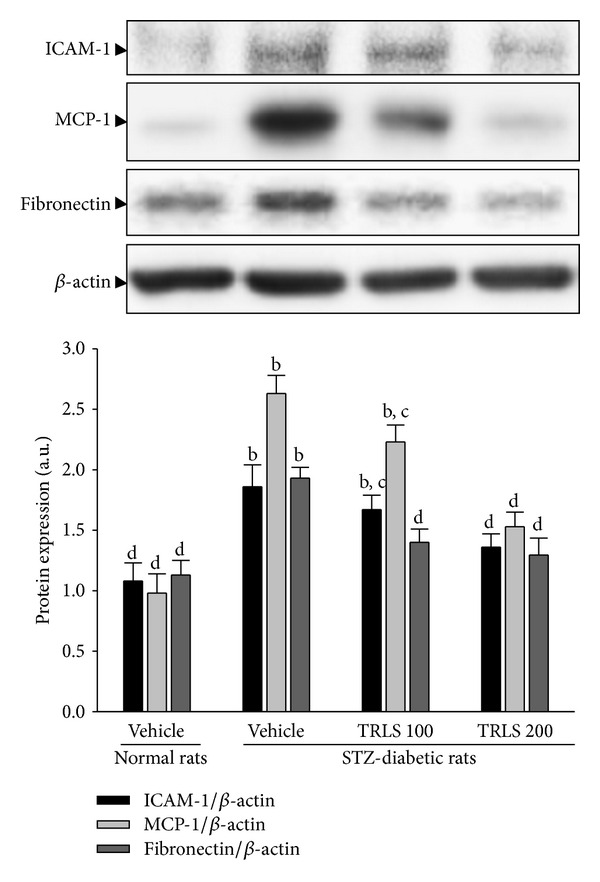
Representative immunoblots of protein expression of ICAM-1, MCP-1, and fibronectin in renal tissues of STZ-diabetic rats treated for eight weeks with TRLS-ext. STZ-diabetic rats were dosed by oral gavage once per day for eight weeks with 100 mg kg^−1^ TRLS-ext (STZ + TRLS 100) or 200 mg kg^−1^ TRLS-ext (STZ + TRLS 200). Normal or STZ-diabetic rats receiving vehicle treatment were administered the same volume of vehicle (distilled water) used to disperse TRLS-ext. Values (mean ± SD) were obtained for each group of 4 animals. ^b^ *P* < .01 compared to vehicle-treated normal rats. ^c^ *P* < .05 and ^d^ *P* < .01 compared to vehicle-treated STZ-diabetic rats, respectively.

The renal ICAM-1 and MCP-1 proteins were 1.8- and 2.6-fold higher in STZ-diabetic rats compared with normal rats, respectively. These increases were ameliorated by 74.2% and 58.7%, respectively, after eight weeks of treatment with TRLS-ext (200 mg kg^−1^ per day) (Figure 3). Renal fibronectin protein levels were also 1.9-fold higher in STZ-diabetic rats relative to normal rats. Treatment of STZ-diabetic rats with 200 mg kg^−1^ TRLS-ext per day for eight weeks resulted in a marked 66.8% reduction of renal fibronectin protein expression compared with that in vehicle-treated counterparts (Figure 3). 

### 3.5. Effect of TRLS-ext on Renal Expression of TNF-*α* and IL-1*β*

**Figure 4:**
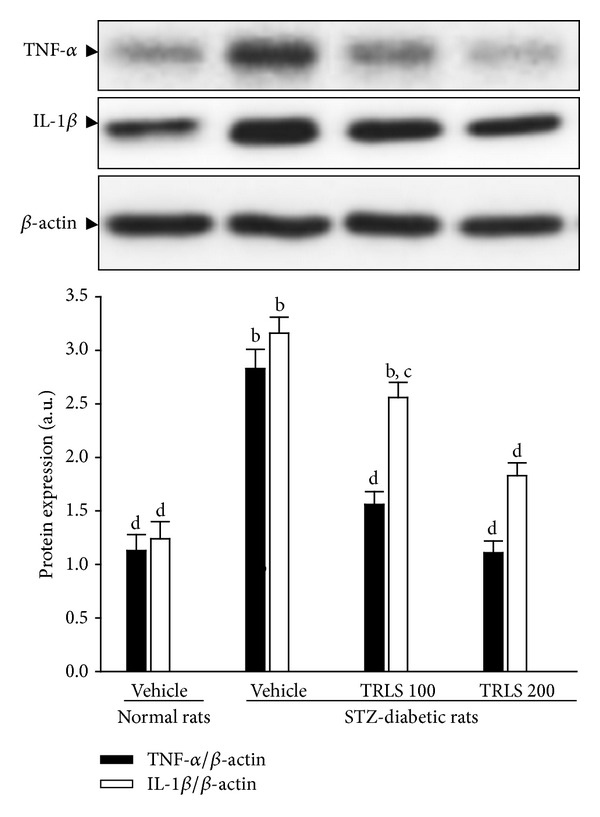
Representative immunoblots of protein expression of TNF-*α* and IL-1*β* in the renal tissues of STZ-diabetic rats treated for eight weeks with TRLS-ext. STZ-diabetic rats were dosed by oral gavage once per day for eight weeks with 100 mg kg^−1^ TRLS-ext (STZ + TRLS 100) or 200 mg kg^−1^ TRLS-ext (STZ + TRLS 200). Normal or STZ-diabetic rats receiving vehicle treatment were given the same volume of vehicle (distilled water) used to disperse TRLS-ext. Values (mean ± SD) were obtained for each group of 4 animals. ^b^ *P* < .01 compared to vehicle-treated normal rats, respectively. ^c^ *P* < .05 and ^d^ *P* < .01 compared to vehicle-treated STZ-diabetic rats, respectively.

Western blot analysis showed that the renal TNF-*α* protein expression was increased 2.5-fold in the STZ-diabetic rats compared with that in the control rats (*P* < .01). A significant increase of renal IL-1*β* protein was also observed in STZ-diabetic rats when compared with the level in the control rats (Figure 4). Treatment for eight weeks with TRLS-ext (200 mg kg^−1^ per day) significantly decreased TNF-*α* protein levels in the kidneys of STZ-diabetic rats to 39.2% of their vehicle counterparts (Figure 4). Renal IL-1*β* protein levels were reduced by 18.9% and 42.1% after daily treatment with 100 and 200 mg kg^−1^ TRLS-ext, respectively, relative to the level in vehicle-treated STZ-diabetic rats (Figure 4).

### 3.6. Effect of TRLS-ext on Renal Expression of I*κ*B*α* and NF-*κ*B

**Figure 5:**
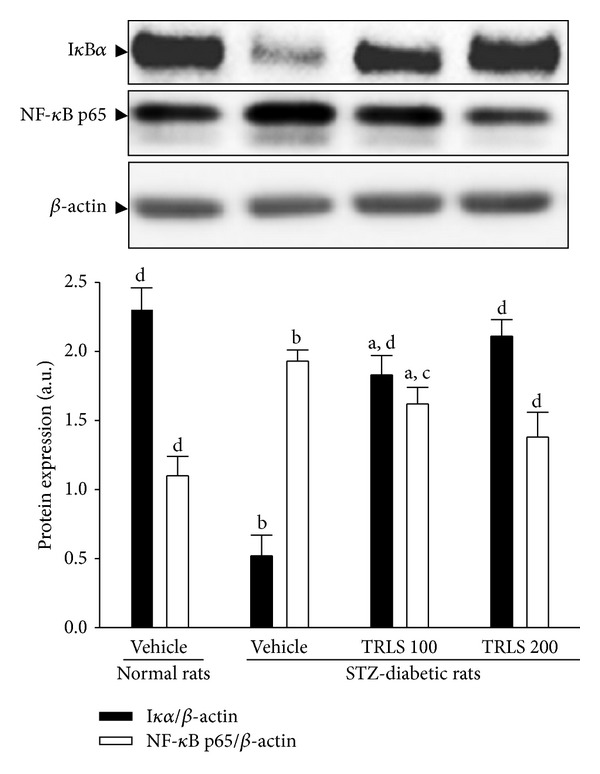
Representative immunoblots of protein expression of I*κ*B*α* and NF-*κ*B in the renal tissues of STZ-diabetic rats treated for eight weeks with TRLS-ext. STZ-diabetic rats were dosed by oral gavage once per day for eight weeks with 100 mg kg^−1^ TRLS-ext (STZ + TRLS 100) or 200 mg kg^−1^ TRLS-ext (STZ + TRLS 200). Normal or STZ-diabetic rats receiving vehicle treatment were given the same volume of vehicle (distilled water) used to disperse TRLS-ext. Values (mean ± SD) were obtained for each group of 4 animals. ^a^ *P* < .05 and ^b^ *P* < .01 compared to vehicle-treated normal rats, respectively. ^c^ *P* < .05 and ^d^ *P* < .01 compared to the values of vehicle-treated STZ-diabetic rats, respectively.

We observed that cytosolic I*κ*B*α* in the kidney of STZ-diabetic rats was reduced to 22.6% of that in the normal group, and TRLS-ext treatment reduced I*κ*B*α* degradation in a dose-dependent manner (Figure 5). Eight weeks of treatment of STZ-diabetic rats with 100 or 200 mg kg^−1^ per day TRLS-ext increased I*κ*B*α* protein levels to 79.6% and 91.7% of that in normal rats, respectively (Figure 5).

STZ treatment significantly increased the NF-*κ*B protein level (by 1.8-fold relative to that of vehicle-treated normal rats) in the kidney (Figure 5). The STZ-induced upregulation of NF-*κ*B protein was reduced 16.1% and 28.6 relative to that in vehicle-treated STZ-diabetic rats after eight weeks of treatment with 100 and 200 mg kg^−1^ TRLS per day, respectively (Figure 5). 

## 4. Discussion

Early DN is characterized by hypertrophy of the glomeruli and tubular epithelial cells, thickening of basement membranes, enhanced renal blood flow, and glomerular hyperfiltration [1]. This is accompanied by increased protein excretion and subsequent development of progressive glomerulosclerosis, accumulation of extracellular matrix proteins in the glomerular mesangium, thickening of glomerular and tubular membranes, and tubulointerstitial fibrosis, all of which contribute to an inexorable progressive deterioration of renal function [1]. In the present study, we observed that treatment with TRLS-ext lowered fasting blood glucose and HbA_1c_ in diabetic rats. Markedly elevated 24-hour levels of BUN, Scr, urine volume, and proteinuria in STZ-diabetic rats were effectively reduced after TRLS-ext treatment. TRLS-ext also caused significant improvements in the relative kidney weight, suggesting that they may reverse kidney hypertrophy in STZ-diabetic rats. The results of our study further indicated that TRLS-ext ameliorated glomerular pathological changes in diabetic kidney. Thus, we demonstrated treatment with TRLS-ext attenuated DN syndrome characterized by proteinuria and the loss of renal function in STZ-diabetic rats. 

Inflammation may be a key factor activated by the metabolic, biochemical, and hemodynamic derangements known to exist in the diabetic kidney. Macrophages are key inflammatory cells mediating kidney inflammation in experimental and human diabetes. In experimental diabetic mice, macrophage accumulation and activation are associated with prolonged hyperglycemia, glomerular immune complex deposition, increased chemokine production, and progressive fibrosis [12, 13]. In human type 2 diabetes, kidney macrophage accumulation is associated with the degree of glomerular sclerosis [18]. Using accumulation of ED-1 as a marker of macrophage activation [19], we have demonstrated that increased macrophage activation in the glomeruli of kidney tissue from STZ-diabetic rats is ameliorated by the administration of TRLS-ext. Activated macrophages elaborate a host of proinflammatory, profibrotic, and antiangiogenic factors. The renal expression of inflammatory cytokines such as TNF-*α* and IL-1*β* was demonstrated to increase in diabetes, contributing to the development of DN [20]. Along with the effects on macrophages, there was a reduction in the upregulated protein expression of TNF-*α* and IL-1*β* from kidneys of STZ-diabetic rats. Thus, we believe that the anti-inflammatory effects of TRLS-ext, through the inhibition of macrophage infiltration, might provide a renoprotective effect in the STZ-induced diabetic model.

ICAM-1 is a cell-surface glycoprotein involved in leukocyte attachment to the endothelium as the ligand for integrin on leukocytes [21]. ICAM-1 is also present on the membranes of macrophages and lymphocytes [21]. ICAM-1 expression is increased in models of type 1 and 2 diabetes [22, 23] and can be induced by hyperglycemia, advanced glycosylation end products, oxidative stress, hyperlipidemia, hyperinsulinemia, and proinflammatory cytokines [24]. Like ICAM-1, MCP-1 is also increased significantly in DN, and expression levels correlate with the number of infiltrating interstitial macrophages. Previous studies have demonstrated that renal MCP-1 is involved in the direction of macrophage migration into the diabetic kidney, while proteinuria, itself, may contribute to this upregulation of MCP-1 [25]. MCP-1 also upregulates the expression of adhesion molecules and promotes the expression of other proinflammatory cytokines [26, 27]. Consistent with these previous reports, we observed that ICAM-1 and MCP-1 expression was increased in experimental diabetic nephropathy and that the increases were attenuated by TRLS-ext treatment. The inhibitory effect of TRLS-ext on MCP-1 and ICAM-1 may be partially due to the decreased infiltration of monocytes/macrophages. Therefore, a possible mechanism for preventing the progression of renal disease may involve the effect of TRLS-ext on attenuating inflammation, by reducing the release of inflammatory mediators or inhibiting the expression of adhesion molecules in the diabetic kidney.

Diabetic mice deficient in MCP-1 and ICAM-1 also show a significant reduction in renal fibrosis with less inflammatory cell infiltration [28], suggesting that the inhibition of inflammatory cell recruitment may lead to a reduction in extracellular matrix accumulation. Our results also show that, along with the decreases in MCP-1 and ICAM-1 expression, TRLS-ext treatment significantly decreases fibronectin expression in the kidneys of STZ-diabetic rats. Therefore, we propose the reduced accumulation of glomerular extracellular matrix in TRLS-ext-treated diabetic rats is a consequence of reduced infiltration of inflammatory cells, in addition to the antifibrotic effect of TRLS-ext.

Transcription factors such as NF-*κ*B regulate the expression of genes for several cytokines and chemotactic and matrix proteins involved in inflammation, as well as regulating immunological responses and cell proliferation [29]. It has been demonstrated that NF-*κ*B is involved in the induction of MCP-1 in mesangial cells cultured under high glucose conditions and that it subsequently mediates macrophage accumulation [30]. NF-*κ*B has been detected in most cell types. It consists of a p50/p65 heterodimer, which is retained in the cytoplasm by the masking of nuclear localization sequence by I*κ*B*α*, the inhibitor of NF-*κ*B [30]. We showed that activation of NF-*κ*B and degradation of I*κ*B were increased in kidneys of STZ-diabetic rats compared with the levels in control rats. TRLS-ext treatment prevented all of these alterations. Treated rats exhibited reduced levels of glucose and HbAc1. All the above results suggest that beneficial effect of TRLS-ext in rats with DN is at least in part through antihyperglycemia which was accompanied by inhibition of macrophage infiltration via reducing NF-*κ*B-mediated inflammatory response.

Previous phytochemical investigations of TRLS have resulted in the isolation of saponins, polysaccharides, and homoisoflavonoids, which have been reported as the major pharmacologically active components [5–7]. A considerable number of saponin glycoside-related compounds such as ruscogenin and ophiopogonin D have been found in TRLS-ext. The specific components of TRLS-ext responsible for its renal-protective effect remain to be identified in future research.

## 5. Conclusion

We have shown that TRLS-ext treatment can ameliorate DN in rat model of type I diabetes through the inhibition of macrophage infiltration in kidney, due to its anti-inflammatory effect. In addition, our results support the findings that TRLS-ext inhibits the activity of NF-*κ*B and the degradation of I*κ*B*α* and, as a result, decreases the expression of proinflammatory cytokines. Enhanced fibronectin expression in the diabetic kidney is also attenuated by TRLS-ext treatment. These findings suggest that TRLS-ext might be considered as potential adjuvant treatment for the prevention of nephropathy in diabetes.

## Figures and Tables

**Table 1:** Biochemical parameters in experimental animals at the end of the eight-week treatment.

	Normal rats	STZ-diabetic rats		
	Vehicle	Vehicle	TRLS-ext 100	TRLS-ext 200
Body weight (BW) (g rat^−1^)	411.16 ± 17.23^d^	267.73 ± 16.47^b^	293.61 ± 16.25^b,c^	342.02 ± 18.47^a,c^
Kidney weight (KW) (g)	1.44 ± 0.21^d^	2.86 ± 0.38^b^	2.05 ± 0.27^a,c^	1.78 ± 0.22^d^
KW/BW ratio (%)	0.35 ± 0.11^d^	1.07 ± 0.13^b^	0.71 ± 0.09^b,c^	0.52 ± 0.08^d^
Plasma glucose (mg dL^−1^)	94.83 ± 7.92^d^	436.29 ± 17.28^b^	352.87 ± 15.26^b,c^	311.26 ± 14.38^b,c^
HbA_1c_ (%)	4.85 ± 1.06^d^	14.61 ± 1.82^b^	12.24 ± 1.47^b^	11.04 ± 1.16^b^
24-h urine volume (mL per day)	8.98 ± 3.12^d^	27.32 ± 4.23^b^	17.67 ± 5.14^b,c^	15.37 ± 4.26^b,c^
24-h urine protein (mg per day)	6.68 ± 3.44^d^	30.72 ± 6.01^b^	16.08 ± 4.02^b,c^	12.02 ± 4.99^b,c^
Scr (*μ*mol L^−1^)	36.21 ± 5.97^d^	90.59 ± 8.47^b^	80.41 ± 7.43^b^	65.18 ± 8.35^b,c^
BUN (mmol L^−1^)	6.72 ± 1.71^c^	17.46 ± 1.93^b^	14.10 ± 2.01^b^	10.78 ± 1.63^a,c^
Ccr (mL min^−1^)	3.92 ± 0.71^d^	1.66 ± 0.82^b^	2.38 ± 0.74^ a,c^	2.76 ± 0.67^d^

STZ-diabetic rats were dosed by oral gavage once per day for eight weeks with 100 mg kg^−1^ TRLS-ext (TRLS-ext 100) or 200 mg kg^−1^ TRLS-ext (TRLS-ext 200). Normal or STZ-diabetic rats receiving vehicle treatment were given the same volume of vehicle (distilled water) used to disperse TRLS-ext. Values (mean ± SD) were obtained for each group of 8 animals. ^a^ *P* < .05 and ^b^ *P* < .01 compared to the values of vehicle-treated normal rats. ^c^ *P* < .05 and ^d^ *P* < .01 compared to the values of vehicle-treated STZ-diabetic rats, respectively.
